# Doing nothing and what it looks like: inactivity in fattening cattle

**DOI:** 10.7717/peerj.9395

**Published:** 2020-07-21

**Authors:** Sara Hintze, Freija Maulbetsch, Lucy Asher, Christoph Winckler

**Affiliations:** 1 University of Natural Resources and Life Sciences, Vienna, Division of Livestock Sciences, Department of Sustainable Agricultural Systems, Vienna, Austria; 2 School of Natural and Environmental Sciences, Newcastle University, Newcastle, United Kingdom

**Keywords:** Animal welfare, Inactive behaviour, Inactivity ethogram, Cattle, Co-occurring postures, Cspade, Machine learning algorithm

## Abstract

**Background:**

Animals kept in barren environments often show increased levels of inactivity and first studies indicate that inactive behaviour may reflect boredom or depression-like states. However, to date, knowledge of what inactivity looks like in different species is scarce and methods to precisely describe and analyse inactive behaviour are thus warranted.

**Methods:**

We developed an Inactivity Ethogram including detailed information on the postures of different body parts (Standing/Lying, Head, Ears, Eyes, Tail) for fattening cattle, a farm animal category often kept in barren environments. The Inactivity Ethogram was applied to Austrian Fleckvieh heifers kept in intensive, semi-intensive and pasture-based husbandry systems to record inactive behaviour in a range of different contexts. Three farms per husbandry system were visited twice; once in the morning and once in the afternoon to cover most of the daylight hours. During each visit, 16 focal animals were continuously observed for 15 minutes each (96 heifers per husbandry system, 288 in total). Moreover, the focal animals’ groups were video recorded to later determine inactivity on the group level. Since our study was explorative in nature, we refrained from statistical hypothesis testing, but analysed both the individual- and group-level data descriptively. Moreover, simultaneous occurrences of postures of different body parts (Standing/Lying, Head, Ears and Eyes) were analysed using the machine learning algorithm cspade to provide insight into co-occurring postures of inactivity.

**Results:**

Inspection of graphs indicated that with increasing intensity of the husbandry system, more animals were inactive (group-level data) and the time the focal animals were inactive increased (individual-level data). Frequently co-occurring postures were generally similar between husbandry systems, but with subtle differences. The most frequently observed combination on farms with intensive and semi-intensive systems was lying with head up, ears backwards and eyes open whereas on pasture it was standing with head up, ears forwards and eyes open.

**Conclusion:**

Our study is the first to explore inactive behaviour in cattle by applying a detailed description of postures from an Inactivity Ethogram and by using the machine learning algorithm cspade to identify frequently co-occurring posture combinations. Both the ethogram created in this study and the cspade algorithm may be valuable tools in future studies aiming to better understand different forms of inactivity and how they are associated with different affective states.

## Introduction

Many of the animals we keep on farms, in labs or in zoos live in monotonous and often barren environments. Under conditions that lack changing stimuli, animals often exhibit active behaviour which is indicative of poor welfare, including abnormal repetitive behaviour (e.g., Würbel, 2001) or re-directed behaviour towards conspecifics (e.g., tail-biting in pigs: Scott et al., 2007). Such behaviours are rather striking and have thus been studied intensively across species. In contrast, relatively little attention has been devoted to the high levels of inactivity often described in animals kept in barren environments (Fureix & Meagher, 2015).

Inactive behaviour is heterogeneous in nature and different forms of inactive behaviour in different contexts may thus have opposite meanings with respect to animal welfare, including the animals’ affective states (as reviewed in Fureix & Meagher, 2015). Being inactive has been described as a potential sign of positive affective states, including relaxation, post-consummatory satisfaction and ‘sun-basking’, but has also been discussed as a potential indicator of negative affective states, including boredom and depression-like states (Fureix & Meagher, 2015).

Most studies so far have focused on inactivity levels in barren compared to enriched environments. Studies with pigs, mice and minks housed in either barren environments (e.g., slatted floor pens for fattening pigs, Bolhuis et al., 2006) or enriched environments (e.g., pen with some sort of substrate, Bolhuis et al., 2006, and extra space, Beattie et al., 2000) found that under barren conditions, animals spend more time being inactive (Beattie et al., 2000; Bolhuis et al., 2006; Fureix et al., 2016; Haskell et al., 1996; Meagher, Campbell & Mason, 2017; Meagher & Mason, 2012). Moreover, pigs and mink experiencing a change from enriched to barren conditions show more inactive behaviour when compared to animals housed in barren conditions (Bolhuis et al., 2006; Meagher et al., 2013). In veal calves, feeding regime can also affect the duration the animals are inactive. Calves being fed a barren diet lie more idle than calves receiving an enriched diet, and calves with *ad libitum* access to straw stand less idle than individuals with no additional straw provided (Webb et al., 2017). In sum, these studies indicate that barren conditions lead to increased levels of inactivity, but whether this inactivity is simply the result of “having nothing else to do” or whether it is indicative of more negative states is largely unknown.

Anecdotally, it has been hypothesised that inactive animals are bored (Wood-Gush & Beilharz, 1983) and that being inactive is a “cut-off” strategy of the animal “to isolate itself from an unsuitable environment” (Pearce, Paterson & Pearce, 1989, p. 35). Only recently, systematic studies on the association between inactive behaviour and animals’ affective states have been conducted. From these studies we know that the level of inactivity in mice tends to predict immobility in the Forced Swim Test, a test used to assess depression in rodents (although the validity of this test has recently been questioned Reardon, 2019). Moreover, “withdrawn” states of inactivity in horses (as defined in Fureix et al., 2012) are associated with anhedonia (Fureix et al., 2015), a key symptom of depression. While these studies indicate a positive relationship between inactivity and depression-like states, others have found no such association (Harvey et al., 2019), or have linked inactive behaviour and boredom-like states. In two independent studies, Meagher and colleagues demonstrated that inactivity in mink is positively associated with some measures of exploration of differently valenced stimuli *a priori* operationally defined as indicating boredom (Meagher, Campbell & Mason, 2017; Meagher & Mason, 2012).

One challenging aspect in the study of inactivity is a lack of consensus on how to define and describe inactive behaviour in different species. Most studies define an animal as being inactive if it is standing or lying (or sitting in the case of pigs), often accompanied by the specification that the animal’s eyes must be open (e.g., Beattie et al., 2000; Meagher & Mason, 2012). However, other definitions exist as well. Bolhuis and colleagues, for example, described pigs as being inactive when lying with eyes either open or closed, but differentiated between these two forms (Bolhuis et al., 2006; Bolhuis et al., 2005). Sitting and standing were not included in the definition of inactivity in these studies. Webb and colleagues differentiated between lying and standing either actively or idle, the latter being described as “displaying no behaviour with an obvious function” (Webb et al., 2017). To our knowledge, only one study has further specified different forms of being inactive (besides distinguishing between standing and lying) by recording three different lying postures in mink (Meagher et al., 2013). Apart from this, detailed descriptions of inactive animals are lacking.

To fill this gap, we aimed to describe inactivity in fattening cattle, *Bos taurus*, an animal category often kept in barren conditions, by developing an Inactivity Ethogram encompassing a description of the animals’ basic body postures, lying postures, head and ear postures as well as the closure of their eyes and tail movements (Objective 1). Especially in the study of inactive behaviour, where overt behaviour is rarely shown, detailed observation of subtle postures of different body parts may reveal important information that are not captured with traditional ethograms. Moreover, we aimed to apply a new tool, the machine learning algorithm “**S**equential **PA**ttern **D**iscovery using **E**quivalence classes” (cspade), to analyse the co-occurrence of the different postures (Objective 2). To this end, we applied the Inactivity Ethogram on farms with intensive, semi-intensive and extensive husbandry systems aiming to describe inactive behaviour of fattening cattle in a range of different contexts.

**Table 1:** Inactivity Ethogram for fattening cattle. Descriptions of all postures per category are given. Postures were continuously recorded when the focal animal was defined as standing or lying inactively.

**Category**	**Posture**	**Description**	**Reference where available**
Basic body postures (Basic)	Standing	Three or four claws on the ground, trunk does not touch the ground, no forward movement	
	Lying	Trunk touches the ground	
	Out of sight	The exact body posture cannot be defined by the experimenter	
Lying postures (Lying)	Lying chest-prone, both front legs under body	Chest-prone position, both front legs are bent at the carpal joint and placed under the body	Sambraus (1971)
	Lying chest-prone, one front leg under body	Chest-prone position, one front leg is bent at the carpal joint and placed under the body, the other front leg is stretched	Haley, Rushen & de Passillé (2000) for definition of the front leg bent (“tucked”) and stretched (“extended”)
	Lying chest-prone, both front legs stretched	Chest-prone position, both front legs are stretched	
	Lying laterally	Either the right or the left side of the trunk on the ground, legs not under the body	Decribed as “lying flat” in Haley, Rushen & de Passillé (2000)
	Out of sight	The exact lying position cannot be defined by the experimenter	
Head postures (Head)	Head up	While standing: head at a height of between the carpal joint and head and back being in a horizontal line; between “head down” and “head raised” While lying: all positions in which the mouth does not touch the ground, the head is not placed on the own body or on a conspecific/an object	
	Head down	While standing: head at a height of between touching the ground and the carpal joint While lying: mouth or other part of the head touches the ground	Haley, Rushen & De Passillé (2000)
	Head raised	Head held above the imaginary horizontal line between the back and the rump	Described as “above horizontal” in Hänninen et al. (2008)
	Head leaned against conspecific	Forehead of focal animal touches body of conspecific	Wierenga & Hopster (1982), Mogensen et al. (1997)
	Head on conspecific/object	Chin or jowl of the focal animal in direct contact with a conspecific or object, e.g., a fence	Described as “head-resting” in Fisher et al. (1997)
	Head on flank	Chin or jowl rests on flank	Tucker et al. (2007)
	Out of sight	The exact head posture cannot be defined by the experimenter	
Ear postures (Ears)	Forwards	Both ears are in or in front of the frontal plane	Described as ears “plane” and “ahead” in Boissy et al. (2011)
	Backwards	Both ears are behind the frontal plane	Described as ears “backward” in Boissy et al. (2011)
	Asymmetrical	Both ears are in two distinct positions relative to the frontal plane, i.e., one ear forwards and one ear backwards	According to ears “asymmetrical” in Boissy et al. (2011)
	Low	Ears hang loosely downwards, fall perpendicular to the head	Described as “EP4” in Proctor & Carder (2014); Gleerup et al. (2015)
	Out of sight	The exact ear posture cannot be defined by the experimenter	
Eye closure (Eyes)	Eyes open	Upper and lower eyelid are not in contact, part of the eyeballs is visible	Not defined, but mentioned in Hänninen et al. (2008)
	Eyes closed	Upper and lower eyelid are in contact, eyeballs invisible	Not defined, but mentioned in Hänninen et al. (2008)
	Out of sight	The experimenter cannot define whether the eyes are open or closed	
Tail movement (Tail)	Tail hanging	Tail hangs straight downwards, minimal soft movements of the lower end of the tail are accepted as the tail still being hanging	
	Tail in motion (tail flinch)	Tail moves from one side to the other, the tip exceeds the height of the focal animal’s knees	
	Out of sight	The experimenter cannot define whether the tail hangs or moves	

## Materials & Methods

## Development of an Inactivity Ethogram

Aiming to capture different aspects of behavioural expression of inactive cattle, we developed an Inactivity Ethogram (Table 1). We first watched existing video clips of fattening cattle to get an impression of the various postures these animals show in order to draft a first ethogram. We then amended our ethogram during four pilot farms visits, during which we observed fattening cattle kept in different husbandry systems ranging from intensive (fully-slatted floor pens) to semi-intensive (feeding and activity area plus straw-bedded lying area) to extensive (pasture). Different types of husbandry systems were chosen to encompass a range of different contexts and thus potentially different forms of inactive behaviour.

Besides amending the ethogram, we also developed a definition for being inactive during our pilot visits. An animal was defined as inactive when it fulfilled two requirements. First, it had to either stand or lie, thus not to change location. Since an animal can also stand or lie in an active manner (e.g., Harvey et al., 2019; Webb et al., 2017), we specified all movements that classified the standing or lying animal as being either inactive or active (Table S1). For movements that could be classified as both, e.g., scratching with one foot, the general rule was that an animal was recorded as active when this movement occurred more than two consecutive times, while movements that were shown once or twice were interpreted as “impulsive” movements, e.g., kicking after flies, and the animal was thus still recorded as being inactive. Second, an animal had to be inactive as defined above for at least 30 s before it was classified as being inactive.

## Application of the Inactivity Ethogram on farms with different husbandry systems

### Experimental design

The Inactivity Ethogram was applied on nine farms in Lower and Upper Austria between April and August 2018. We visited three farms per husbandry system (intensive, semi-intensive, pasture) and all farms were visited twice, resulting in 18 farm visits. Data were recorded between 9:30 AM and 06:00 PM, with one visit per farm taking place in the morning (and lasting for approximately five hours) and the other one in the afternoon (again for approximately five hours). We thus covered most of the daylight hours and consequently potentially different forms of inactivity. The order of farm visits was counterbalanced across husbandry system and time of visit (morning/afternoon). All nine farmers agreed to take part in the study, signed an informed consent form and approved the use of data from their farms for research purposes within the context of this study.

During each farm visit, we took 16 video clips of 15 min each with a Panasonic HDC-SD99 or a JVC GZ-R410BEU camcorder mounted on a tripod. Whenever possible, the 16 video clips were taken from 16 different pens on intensive and semi-intensive farms; on farms with fewer than 16 pens, we first recorded all existing pens before starting with the first pen again. The order of recorded pens was reversed during the second visit. After recording of the first eight pens, there was a break of approximately one hour before the second half of the pens was recorded. On farms with pasture, the same group of cattle was recorded for the same duration as on intensive and semi-intensive farms, i.e., for a total of four hours.

Simultaneously to the camera recording, one focal animal of the recorded pen/group was continuously observed for 15 min. For a focal animal to be chosen, it had to be defined as being inactive for at least 30 s prior to the start of observation. Moreover, focal animals were selected based on predefined rules to avoid a biased selection of animals by balancing for distance between focal animal and observer and for the position of the focal animal within its group. Lame and obviously sick animals were not chosen as focal animals. Data were recorded live using Mangold INTERACT^®^ (light version 17.1.11.0) on a Microsoft tablet (Acer Iconia W510). Animals were observed live to be able to record more of the details we were interested in, e.g., whether an animal’s eyes were open or closed, which we could not detect on the video clips taken during our pilot visits. Previous studies of inactivity used live observations as well, e.g., in mink (Meagher, Campbell & Mason, 2017; Meagher & Mason, 2012), mice (Fureix et al., 2016), horses (Fureix et al., 2012) and in a study recording ear, neck and tail postures in dairy cattle (De Oliveira & Keeling, 2018); however, we cannot fully rule out that the presence of the observer affected the animals’ behaviour. During the observations, the observer stood on a ladder (feet approximately 80 cm above the ground and two to three meters away from the pens/pasture) to have a better view into the pen, e.g., for focal animals further back in the pen or on pasture. Whenever needed, especially on pasture, she used a binocular.

### Farms

We visited three farms with fully-slatted floor pens (INTENSIVE), three farms with a feeding area, an activity area and a lying area bedded with straw (SEMI) and three farms where the animals were kept on pasture (PASTURE); one farm with daytime pasture and two farms with access to the pasture during day and night (Table S2, Fig. S1).

### Animals

All focal animals were female Austrian Fleckvieh, a dual-purpose breed, or Fleckvieh crosses while non-focal animals were sometimes of different breeds (e.g., Limousin, Belgian Blue) and crossbreeds. We only observed heifers since bulls are very rarely kept on pasture in Austria and we aimed to avoid confounding sex and husbandry system. Heifers ranged between 8 and 27 months in age and weighed at least 300 kg according to the farmers’ estimations.

## Data preparation for analysis

### Group level data from the video recordings

Inactivity at the pen/group level was analysed in all 288 15-minute video clips (18 farm visits x 16 video clips per farm). Scan samples were conducted on still images at minutes 2, 7 and 12, and the number of inactive animals as well as the number of inactively lying animals was recorded (*n* = 858 scans, 288 video clips x 3 scans per clip with six scans missing due to camera failure, “group sample datasheet”). An animal was recorded as inactive if it fulfilled the behavioural criteria for being inactive as described above for at least 30 s prior to the time when the still image was analysed to ensure that the same definition of inactivity was applied on the group and the individual level. Data derived from 86 (farm visit 1) and 88 animals (farm visit 2) on INTENSIVE, 151 (farm visit 1) and 152 (farm visit 2) on SEMI and 60 animals per farm visit on PASTURE farms. Video clips were analysed in random order by an observer who was not involved in this study otherwise. She was blind with respect to the farm, the number of visit (first or second) and the time of visit (morning or afternoon), but not to the husbandry system which was identifiable on the video clips.

### Focal animal data from the live observations

Data from the live observations were exported from Mangold INTERACT^®^ in two different formats. For a descriptive overview of the data, the total duration of being inactive and per posture while being inactive was exported for each animal (*n* = 288, “individual duration datasheet”). Moreover, data of all postures while being inactive were extracted per second, i.e., the continuously recorded data were translated to one-second interval samples. As a result, we received information of the postures per body part and sampling point (i.e., seconds) allowing us to analyse which postures occurred simultaneously. Only sampling points that yielded complete information of the basic body postures, head, ear, eye and tail were considered. Sampling points for which one or more of the postures had been recorded as “out of sight” were discarded (“individual sample datasheet”).

## Statistical analyses

Since our study is explorative, we refrained from formal hypothesis testing, but instead present our data descriptively. The description of the co-occurrence of the different postures is based on analysis with the machine learning algorithm cspade.

## Scan samples on the group level

For a descriptive overview, group-level data are presented as the mean percentage (± standard deviation) of animals being inactive and of animals lying while being inactive per pen/group and per sampling point (*n* = 858 sampling points). To this end, means of the three scans per observation were calculated.

## Continuous observations on the focal animal level

## a) Percentage of time the single postures were observed for.

The description of the total time the animals spent inactive is based on the “individual duration datasheet”, while all other outcome measures are described based on the “individual sample datasheet”. We divided the number of sampling points (i.e., seconds) for each posture (e.g., “Ears forward”) by the total number of sampling points for the respective body part (i.e., all sampling points for which Ears were recorded). This allowed to account for differences between animals in the number of sampling points (since the postures were only recorded when the focal animal was inactive) and for the number of sampling points the respective body part (e.g., Ears) was “out of sight”.

## b) Simultaneous occurrences of postures of different body parts.

The analyses of the simultaneous occurrences of postures are based on the “individual sample datasheet”. We included whether the animals are standing or lying (Basic), their head postures (Head), ear postures (Ears), and the closure of the eyes (Eyes). Tail movement was excluded since tail was recorded as “hanging” for most of the time and was in motion almost exclusively on PASTURE. To understand patterns in simultaneous observation of postures in a manner, which accounts for frequency of occurrence within as well as between animals, a machine learning algorithm was used. The **S**equential **PA**ttern **D**iscovery using **E**quivalence classes (cspade) algorithm (Zaki, 2001) was used for this purpose in R (version 3.6.0, package: arulesSequences, function: cspade; Buchta & Hahsler, 2019). This algorithm is commonly used to identify which items are purchased together in shopping baskets or in subsequent shopping trips (Koenecke, 2019). When using this algorithm, the support threshold was set at 0.1 and the maximum gap (maxgap) was set at 0 to search only for postures that occurred simultaneously (and not in subsequent observations). Support is the proportion of a given combination out of the total number of co-occurring combinations. The support threshold is the value above which co-occurring combinations are identified as frequent combinations. The algorithm outputs the confidence for given co-occurring combinations, where confidence is the likelihood of observing that combination, in this case the combination of body parts, in future observations. This is calculated by the conditional probability of observing the combination given it has already been observed in that animal. The cspade algorithm was calculated separately for each husbandry system. To account for the fact that the different husbandry systems had different numbers of total observations and different numbers of animals, we compared cspade results from full data to data truncated to account for this (reduced so that husbandry systems matched for number of observations and number of animals in a randomised manner). Since truncated data and full datasets were not different (correlation *r*>0.99), we present data from the full datasets here. The confidence of pairwise combinations of body parts (frequent sequences of two) and all four body parts is displayed, the former as a network figure with confidence used as the strength of connections between body parts (using visNetwork and igraph). Differences in confidence noted between husbandry systems are presented and since several studies defined animals as being inactive if they were immobile with eyes open (Meagher & Mason, 2012; Fureix et al., 2016), we also focus on presenting the confidence values for the pairwise combinations “Lying with Eyes open”, “Lying with Eyes closed”, “Standing with Eyes open” and “Standing with Eyes closed”.

**Figure 1:**
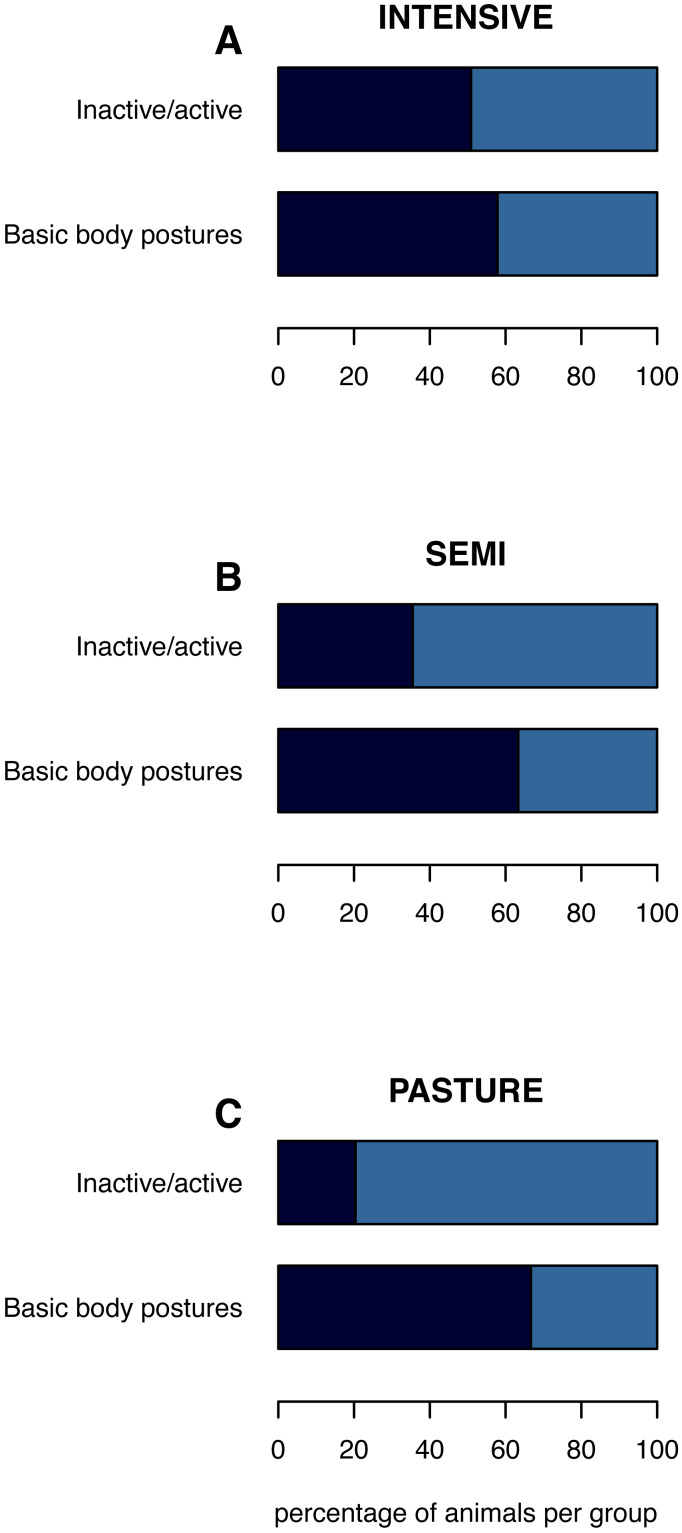
(In)activity levels and basic body postures on the group level. The mean percentage of inactive animals per group and of inactively lying or standing animals is shown per husbandry system: (A) INTENSIVE, (B) SEMI, (C) PASTURE. **Inactive/active:** inactive (navy), active (azure). **Basic body postures:** lying (navy), standing (azure) while being inactive.

**Figure 2:**
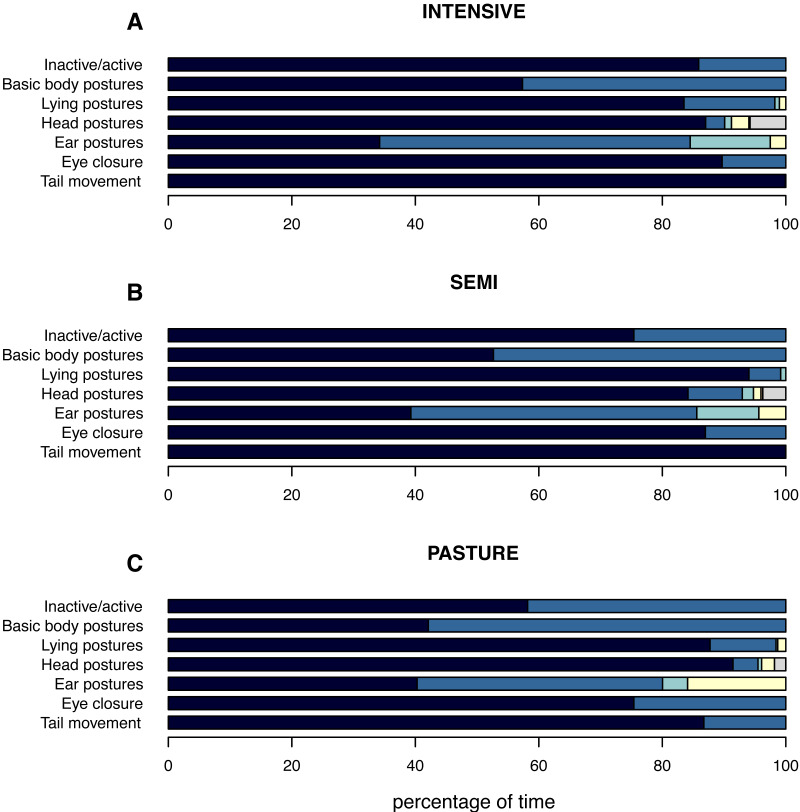
(In)activity levels and postures displayed by the focal animals while being inactive. The mean percentage of time the focal animals spent inactive and showed the different postures while being inactive is shown per husbandry system: (A) INTENSIVE, (B) SEMI, (C) PASTURE. **Inactive/active:** inactive (navy), active (azure). **Basic body postures:** lying (navy), standing (azure). **Lying postures:** two front legs under body (navy), one front leg under body (azure), both front legs stretched (turquoise), lateral (yellow). **Head postures:** up (navy), down (azure), raised (turquoise), on conspecific (yellow), leaned (orange), on own body while lying (grey). **Ear postures:** forwards (navy), backwards (azure), asymmetrical (turquoise), low (yellow). **Eye closure:** open (navy), close (azure). **Tail movement:** no movement (navy), movement (azure).

## Results

### Scan samples on the group level

The percentage of inactive animals per pen/group was lowest on PASTURE (mean ± standard deviation: 20.4% ± 31.9), intermediate on SEMI (35.5% ±26.1) and highest on INTENSIVE farms (50.9% ± 29.2; Fig. 1). When being inactive, the percentage of inactively lying animals per group was 66.7% (±39.4) on PASTURE, 63.3% (±38.6) on SEMI and 57.9% (±39.6) on INTENSIVE farms.

### Continuous observations on the focal animal level

#### Percentage of time the single postures were observed for

The pattern of the focal animals’ inactivity level corresponded to the inactivity level on the group level with cattle spending least time inactively on PASTURE (55.8% ± 40.0), followed by SEMI (75.0% ± 27.1) and INTENSIVE farms (85.5% ± 20.7; Fig. 2). When related to the total time spent inactive, the percentage of inactive lying was on average highest on INTENSIVE (57.4% ± 47.6), intermediate on SEMI (52.7% ± 47.3) and shortest on PASTURE farms (42.1% ± 45.7). However, independent of husbandry system, animals spent almost twice as long lying inactively than standing inactively (INTENSIVE: lying a : 55.3%, standing: 30.2% a ; SEMI: lying: 48.6%, standing: 26.4%; PASTURE: lying: 35.6%, standing: 20.2%).

When lying, a chest-prone position with the two front legs tucked under the body was observed for the longest (88.3% ± 27.2 across husbandry systems) and the most commonly observed head position across all husbandry systems was the head held up (87.3% ± 19.3). Cattle on INTENSIVE and SEMI farms showed ears backwards for the longest (INTENSIVE: 50.9%±27.8; SEMI: 47.1% ± 30.1), followed by ears forward (INTENSIVE: 33.8% ± 27.5; SEMI: 38.6% ± 29.5), while these two ear postures were displayed almost equally long by cattle on PASTURE (backwards: 40.0% ± 31.7, forwards: 39.7% ± 31.3). The percentage of time cattle displayed low ears was shortest on INTENSIVE (2.4% ± 8.4), intermediate on SEMI (4.4% ± 13.0) and highest on PASTURE farms (16.2% ± 28.9), whereas the pattern for the time their ears were in an asymmetrical position was the opposite (PASTURE: 4.1% ± 12.5, SEMI: 9.9% ± 12.2, INTENSIVE: 12.9% ± 14.3). On INTENSIVE and SEMI farms, the animals’ eyes were open for almost the same percentage of time (INTENSIVE: 88.1% ± 28.7, SEMI: 87.8% ± 33.0), while they were open for 75.5% (±36.6) of the inactivity time in cattle on PASTURE. As described above, the tail was hanging for almost all of the inactivity time (more than 99%) on INTENSIVE and SEMI farms, while it was recorded as moving for 13.4% (±25.2) of the time in cattle on PASTURE. To indicate which postures could be observed more or less easily in the different husbandry system, the mean percentage of “out of sight” per posture is given in Table S3.

#### Simultaneous occurrence of postures of different body parts

##### a) Overview.

For analyses of the simultaneous occurrences of different postures, the final dataset comprised 131,791 time points (i.e., seconds), corresponding to 36.6 h of observation, for which we had information of all four body parts (Basic, Head, Ears, Eyes; tail movements were excluded as described above). It did not include 17 animals for which full information of all four body parts was not available (INTENSIVE: *n* = 0, SEMI: *n* = 1, PASTURE: *n* = 16), leading to a total of 271 animals in the final dataset. The number of data points per animal varied greatly between individuals and was highest in INTENSIVE, followed by SEMI and PASTURE (INTENSIVE: 565 ± 268, SEMI: 451 ± 291, PASTURE: 434 ± 337).

To ensure that data were not skewed by single individuals displaying the same combination of postures for a long time, we used the cspade algorithm which accounts for the frequency of occurrences both within and between animals and used the confidence, i.e., the likelihood of observing the respective combination of postures displayed in future observations, from this algorithm as outcome measure (ranging from 0 to 1). Between 75 (PASTURE) and 92 (SEMI, INTENSIVE) posture combinations were identified as co-occurring frequently (Table 2).

##### b) Pairwise combinations of postures.

**Figure 3:**
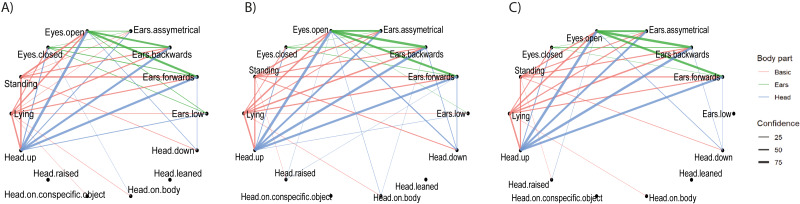
Pairwise combinations of postures. The relationship between pairwise combinations of postures (frequent sequences of two) of Basic, Head, Ears and Eyes are shown per husbandry system: (A) INTENSIVE, (B) SEMI, (C) PASTURE. The thickness of the lines indicates the confidence values of the combination of postures calculated using the cspade algorithm. The colours represent the involved body parts; **red:** Basic plus Head, Ear or Eye; **blue:** Head plus Ear or Eye; **green:** Ear plus Eye.

The most frequently co-occurring combinations of postures of two body parts (e.g., Basic plus Ear, Head plus Eye) across individuals were similar between husbandry systems (Fig. 3). The combination with the highest confidence was “Head up with Eyes open” for all husbandry systems. A small number of combinations were identified as frequently occurring in one or two husbandry systems only. For example, “Ears low” co-occurred with “Lying”/“Head up”/“Eyes open” and “Eyes closed” frequently enough to be displayed only on PASTURE, co-occurred with the same postures but not with “Eyes closed” in SEMI and did not co-occur with any posture in INTENSIVE. Conversely, “Head raised” only co-occurred with other postures in INTENSIVE and SEMI, but not on PASTURE. These combinations tended to be those with the lowest confidence values, which were just above the cut-off threshold. “Lying with Eyes open” as a combination had a lower confidence on PASTURE, particularly when compared to INTENSIVE, whereas it was the opposite for “Standing with Eyes open” (Table 3).

**Table 2:** Number of frequent combinations. The number of frequent combinations as identified by the cspade algorithm for pairwise (e.g., Basic plus Head, Head plus Eye), threefold (e.g., Basic plus Head plus Ear, Head plus Ear plus Eye) and fourfold combinations (Basic plus Head plus Ear plus Eye) as well as the sum of all frequent combinations is presented per husbandry system.

	**Two body parts**	**Three body parts**	**Four body parts**	**Total of combinations**
INTENSIVE	39	40	13	92
SEMI	37	40	15	92
PASTURE	32	32	11	75

**Table 3:** Confidence values for Lying and Standing with Eyes open and Eyes closed. Confidence values of the pairwise combinations of Lying and Standing with Eyes open and Eyes closed identified using the cspade algorithm are presented per husbandry system. Confidence values (likelihood of observing the respective combination of postures displayed in future observations) can range between 0 (not likely) and 1 (very likely).

	**Lying with Eyes open**	**Lying with Eyes closed**	**Standing with Eyes open**	**Standing with Eyes closed**
INTENSIVE	0.61	0.40	0.52	<0.01
SEMI	0.59	0.32	0.53	0.05
PASTURE	0.55	0.38	0.64	<0.01

##### c) Fourfold combinations of postures.

**Figure 4:**
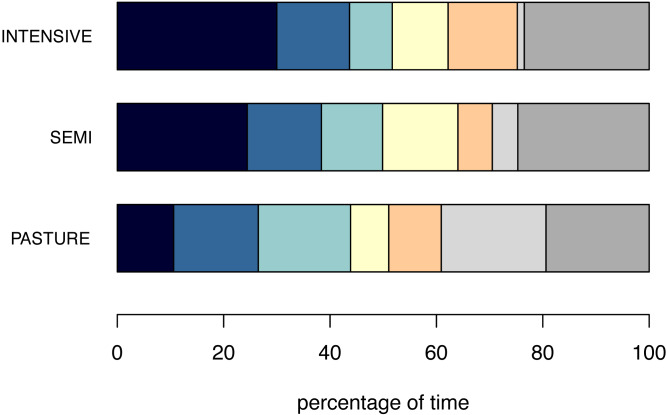
Fourfold combinations of postures. The mean percentage of time the animals spent displaying the six most common fourfold combinations of postures (combinations displayed for at least 5% of the inactivity time across husbandry systems) are shown per husbandry system. From left to right: Lying_Head up_Ears backwards_Eyes open (navy), Standing_Head up_Ears forwards_Eyes open (azure), Lying_Head up_Ears backwards_Eyes closed (turquoise), Lying_Head up_Ears forwards_Eyes open (yellow), Standing_Head up_Ears backwards_Eyes open (orange), Lying_Head up_Ears low_Eyes closed (light grey), all other combinations (dark grey).

The fourfold combination shown for the largest percentage of time on INTENSIVE and SEMI farms was “Lying with Head up, Ears backwards and Eyes open”, whereas it was “Lying with Head up, Ears low and Eyes closed” on PASTURE (Fig. 4). The confidence values from the cspade analysis, which accounts for frequencies both within and between individuals, was also highest for the combination “Lying with Head up, Ears backwards and Eyes open” for INTENSIVE (0.604, Table 4) and SEMI farms (0.568). However, the highest confidence value on PASTURE farms was 0.575 for the combination “Standing with Head up, Ears forwards and Eyes open”. Of the frequently occurring fourfold combinations of Basic, Head, Ears and Eyes, ten were the same across all husbandry systems (confidence ≥ 0.1 for all three husbandry systems, Table 4).

**Table 4:** Confidence values for fourfold combinations of postures. Confidence values for frequent combinations of postures of all four body parts (Basic, Head, Ears, Eyes) identified using the cspade algorithm are given per husbandry system. Confidence values (likelihood of observing the respective combination of postures displayed in future observations) can range between 0 (not likely) and 1 (very likely). Only combinations with a confidence value ≥0.1 are shown. Combinations are ordered from the largest to the smallest maximum confidence value across husbandry systems. For each combination, the largest confidence value is marked in dark blue, the second smallest in medium blue, the smallest in light blue and values less than 0.1 in white.

**Body part combination**	**INTENSIVE**	**SEMI**	**PASTURE**
Lying_Head up_Ears backwards_Eyes open	0.604	0.568	0.363
Standing_Head up_Ears forwards_Eyes open	0.490	0.484	0.575
Standing_Head up_Ears backwards_Eyes open	0.479	0.400	0.425
Lying_Head up_Ears forwards_Eyes open	0.438	0.442	0.375
Standing_Head up_Ears asymmetrical_Eyes open	0.417	0.411	0.175
Lying_Head up_Ears asymmetrical_Eyes open	0.406	0.326	0.088
Lying_Head up_Ears backwards_Eyes closed	0.333	0.284	0.338
Standing_Head down_Ears forwards_Eyes open	0.198	0.295	0.200
Lying_Head up_Ears forwards_Eyes closed	0.188	0.200	0.275
Standing_Head down_Ears backwards_Eyes open	0.188	0.211	
Lying_Head up_Low ears_Eyes closed		0.105	0.200
Lying_Head up_Low ears_Eyes open		0.116	0.163
Lying_Head up_Ears asymmetrical_Eyes closed	0.156	0.126	0.138
Lying_Head on body_Ears backwards_Eyes open		0.137	
Standing_Head raised_Ears forwards_Eyes open	0.115	0.126	
Lying_Head on conspecific or object_Ears backwards_Eyes open	0.104		

## Discussion

The aims of our study were to develop an Inactivity Ethogram for fattening cattle including subtle postures (Objective 1) and to describe co-occurring postures of different body parts recorded with this ethogram on farms with different husbandry systems (Objective 2). Using the machine learning algorithm cspade, we found that the general pattern of co-occurring postures was relatively similar across husbandry systems, but with subtle differences.

Our Inactivity Ethogram encompasses various postures of different body parts and may be a promising methodological tool in future studies investigating different kinds of inactive behaviour. Especially in conditions where the valence of the animals’ inactivity is specifically manipulated and/or investigated, the application of our Inactivity Ethogram may be used to help differentiate between positive inactivity, for example reflecting relaxation, and negative inactivity, for example reflecting boredom or depression-like states. Detailed observation of the postures of different body parts is especially useful in the study of inactive behaviour, where overt behaviour is rarely shown and subtle differences in postures are not captured with traditional ethograms. Recording of animals’ postures and the simultaneous occurrence of postures of different body parts may also have more general application to the study of affective states. De Oliveira & Keeling (2018), for example, already stressed the importance of capturing details of the animals’ postures, when they investigated ear and neck positions as well as tail movements of dairy cows exposed to three routine situations to identify indicators of affective valence.

Since our Inactivity Ethogram was developed and applied on farms with different husbandry systems, it may be used in a range of contexts. We only observed Fleckvieh heifers in our study, however, the general nature of the ethogram means it is likely to be applicable to other cattle breeds or categories such as bulls, and may even be easily adapted for other species.

The number of animals being inactive per group and the percentage of time focal animals were inactive was lowest on PASTURE, intermediate on SEMI and highest on INTENSIVE farms. However, the results need to be interpreted carefully because of the large standard deviations and because we could not account for dependencies in the descriptive analysis. The general pattern is in line with other studies that show increased inactivity levels in barren compared to enriched housing conditions in pigs (Bolhuis et al., 2006; Bolhuis et al., 2005), mink (Meagher & Mason, 2012) and mice (Fureix et al., 2016). However, we did not specifically compare barren and enriched conditions, but whole husbandry systems that differed in many more aspects than just barrenness. Moreover, housing conditions in our study differed between farms whereas the previous studies were conducted in an experimental setting, in which the conditions were manipulated within farm.

The percentage of inactively lying animals on the group level was highest on PASTURE, intermediate on SEMI and lowest on INTENSIVE farms. This is on first glance contradictory to the average percentage duration individual animals spent lying while being inactive. However, two aspects need to be considered here. First, the ratio between inactively lying and inactively standing animals with respect to the total observation time was comparable across husbandry systems with animals spending almost double the time lying inactively than standing inactively. Second, we did not balance for standing and lying animals when selecting our focal animals (and switches between standing and lying in the course of the observation only occurred in 53 of the 288 focal animals). More standing animals were selected on SEMI (50 out of 96) and PASTURE farms (46 out of 86; 10 animals became active immediately after having been chosen) whereas more lying animals were selected on INTENSIVE farms (53 out of 96). This unbalanced selection of focal animals might have skewed the results on the focal animal level and we thus suggest to balance for the basic body position (standing, lying) in future studies.

Even though analysis was descriptive, our study gives insight into potentially interesting patterns (but the same caution as with the group level data applies here to the average durations of the individual data). Ears held asymmetrically, for example, were shown for the shortest on PASTURE, intermediate on SEMI and longest on INTENSIVE farms, while it was the exact opposite for the occurrence of low ears (PASTURE >SEMI >INTENSIVE). Other studies on ear postures might have served for a tentative interpretation of our findings, but, unfortunately, the results from the existing studies are quite inconsistent. For example, one of the most commonly shown ear postures in this and other studies, ears backwards, has been described as reflecting both a positive state (e.g., De Oliveira & Keeling, 2018; Proctor & Carder, 2014) and a negative state (e.g., Boissy et al., 2011 in sheep; Gleerup et al., 2015). Moreover, low ears have been described to be indicative of positive low arousal states (Proctor & Carder, 2014; described as “ears loosely hung down”), but also as a sign of pain in dairy cattle (Gleerup et al., 2015). For the asymmetrical ears, De Oliveira & Keeling (2018) differentiated between right and left asymmetry depending on which ear was pointing backward, and predicted based on their results that right ear backwards might reflect a more positively valenced state than left ear backwards, in accordance with the concept of emotional lateralisation (as reviewed in Leliveld, Langbein & Puppe, 2013). We did not differentiate between left and right ear when recording asymmetry but such a differentiation may be a valuable addition to our Inactivity Ethogram in future studies. It is also important to keep in mind that other factors than the animals’ emotional states, e.g., the level of noise on a farm, might explain differences in ear postures.

Cows’ eyes were open for almost 90% of the inactivity time in INTENSIVE and SEMI, and for approximately 75% of the inactivity time on PASTURE. Closed eyes may indicate more sleep on pasture. This would be in line with findings that cattle on pasture have several episodes of rest and sleep during the day (Sambraus, 1971) and that cattle kept indoors mostly sleep at night (Ternman et al., 2019). However, it is important to note that the closure of the eyes alone is not sufficient to define the animals as being asleep and other characteristics that differentiate between sleep and resting (Nicolau et al., 2000) were not recorded in our study.

We only included two tail postures in our ethogram since a more detailed recording of the tail was not possible during live observations. As a binary outcome measure, tail posture was possibly not recorded in sufficient detail to capture potential differences between contexts. However, “tail hanging stationary” was the most frequent tail position in our study and in De Oliveira & Keeling (2018) where more categories were recorded. Movement of the tail in our study was almost only recorded on PASTURE farms, where it was likely displayed to flick away flies (Dougherty et al., 1995).

By analysing co-occurring postures of different body parts with the cspade algorithm, we detected the combinations of postures that were shown most frequently while animals were inactive. The number of distinct detected combinations was higher for INTENSIVE and SEMI than for PASTURE. One would expect animals in more intensive systems to show fewer different postures in line with studies showing that behavioural diversity is reduced in barren compared to enriched systems (e.g., Haskell et al., 1996; Powell, 1995; Wemelsfelder et al., 2000). However, these studies focused on active behaviour, and not on co-occurring postures. It is thus possible that behavioural diversity is increased in enriched conditions with respect to active but not inactive behaviour.

We specifically investigated the pairwise combinations of Lying and Standing with either Eyes open or closed due to previous studies defining inactivity as the animal being still but awake with eyes open. Based on cspade confidence values, “Lying” and “Eyes open” more frequently co-occurred in INTENSIVE, followed by SEMI and PASTURE, in line with studies which found animals to be more still with eyes open in barren compared to enriched conditions (e.g., Fureix et al., 2016; Meagher, Campbell & Mason, 2017; Meagher & Mason, 2012). The opposite pattern was observed for “Standing” and “Eyes open”, which co-occurred more on PASTURE, followed by SEMI and INTENSIVE. This result indicates the potential importance of distinguishing between different basic body postures and the simultaneous closure of the eye. Cattle on INTENSIVE farms were “Lying with Eyes open” and “Lying with Eyes closed” more frequently, which is in accordance with Bolhuis and colleagues (2005) who found fattening pigs spending more time lying with both open and closed eyes in barren compared to enriched conditions. This result indicates that lying with eyes open goes along with more lying overall, but our finding needs to be interpreted carefully. First, the comparably higher value for animals from INTENSIVE farms does not necessarily mean that the same individuals accounted for both “Lying with Eyes open” and “Lying with Eyes closed”. Second, species-specific differences need to be taken into account. While being still with eyes open may be a good indicator of boredom or depression-like states in mink and mice, respectively, cattle spend about seven hours a day ruminating (Beauchemin, 2018) either with eyes open or closed, which is why lying or standing with eyes open should not *per se* be interpreted as a sign of a negative state in cattle.

The six most frequently co-occurring fourfold combinations (i.e., Basic + Head + Ears + Eyes) were shown for approximately three quarters of the inactive time, while the remaining four fourfold combinations plus the combinations that did not reach the threshold of 0.1 accounted for the remaining 25% of the time. The pattern was relatively similar for INTENSIVE and SEMI, but differed for PASTURE, where the most frequently observed combination (Lying with Head up, Ears low and Eyes closed) did not correspond to the combination with the highest confidence value from the cspade analysis (Standing with Head up, Ears forwards and Eyes open). Lying with Head up, Ears low and Eyes closed was displayed by only 15 individuals (from 96 animals on PASTURE), while Standing with Head up, Ears forwards and Eyes open was displayed slightly less frequently, but by 33 animals. The machine learning algorithm cspade accounts for frequencies both within and between animals, which is why the confidence value was higher for Standing with Head up, Ears forwards and Eyes closed than for Lying with Head up, Ears low and Eyes closed.

Cspade is a promising statistical tool to describe behavioural data. With the constant increase in automatically recorded data, for example by accelerometers (e.g., Stewart et al., 2017), an algorithm like cspade may help analysing information simultaneously obtained from different sensors/data loggers of the same animal over many time points. In addition to the analysis of co-occurrences of postures of different body parts, it would be valuable to analyse transitions between postures in future studies, since behaviour is not a snap shot in time, but dynamic (Asher et al., 2009). Specifically, we suggest analysing transitions per body part (e.g., transitions between different ear postures) as well as transitions between the simultaneous occurrences of postures from different body parts (e.g., from “Standing with Head up, Ears forwards, Eyes open” to “Lying with Head up, Ears backwards, Eyes open”) to provide a more complete picture of the whole animal and thus to advance our understanding of what different forms of inactive behaviour mean for animal welfare.

## Conclusions

Our study is the first to explore inactivity in fattening cattle. The Inactivity Ethogram to capture subtle postures and the machine learning algorithm cspade to describe co-occurring postures of different body parts may be valuable tools for future studies on the welfare relevance of inactive behaviour.

##  Supplemental Information

10.7717/peerj.9395/supp-1 Supplemental Information 1 Pictures illustrating the three husbandry systems On INTENSIVE farms heifers were kept in pens with fully slatted floor, on SEMI farms they were provided with a straw bedded lying area and on PASTURE farm heifers spent either the day or day and night on pasture.

10.7717/peerj.9395/supp-2 Supplemental Information 2 Movements classifying standing and lying animals as being inactive or being active Note that some movements could be classified as both the animal still being inactive and the animal becoming active based on the number of times this movement was shown (up to two consecutive times: classified as still being inactive, more than two consecutive times: classified as becoming active).

10.7717/peerj.9395/supp-3 Supplemental Information 3 Overview of some farm characteristics per husbandry system

10.7717/peerj.9395/supp-4 Supplemental Information 4 “Out of sight” for the different categories of the Inactivity Ethogram The mean percentage of time (some parts of) the focal animals were recorded as “Out of sight” with respect to the different categories of the Inactivity Ethogram is presented per husbandry system.

10.7717/peerj.9395/supp-5 Supplemental Information 5 Group sample datasheet

10.7717/peerj.9395/supp-6 Supplemental Information 6 Individual duration datasheet

10.7717/peerj.9395/supp-7 Supplemental Information 7 Individual sample datasheet

## Additional Information and Declarations

### Field Study Permissions

The following information was supplied relating to field study approvals (i.e., approving body and any reference numbers): All nine farmers initially agreed to take part in the study, signed an informed consent form and approved the use of data from their farms for research purposes within the context of this study.

### Data Availability

The following information was supplied regarding data availability: Data is available at Figshare: - Group level datasheet: Hintze, Sara (2019): Inactivity in cattle_Group sample datasheet.xlsx. figshare. Dataset. https://doi.org/10.6084/m9.figshare.10264925.v5. - Individual duration datasheet: Hintze, Sara (2019): Inactivity in cattle_Individual duration datasheet. figshare. Dataset. https://doi.org/10.6084/m9.figshare.10264931.v4. - Individual sample datasheet: Hintze, Sara (2019): Inactivity in cattle_Individual sample datasheet.xlsx. figshare. Dataset. https://doi.org/10.6084/m9.figshare.10264943.v4.
