# Supplementary material for: Doing nothing and what it looks like: inactivity in fattening cattle
Source: PeerJ. 2020 Jul 21;8:e9395. doi: 10.7717/peerj.9395 (PMC7512136; doi:10.7717/peerj.9395)
Supplement: Supplemental Information 1 — On INTENSIVE farms heifers were kept in pens with fully slatted floor, on SEMI farms they were provided with a straw bedded lying area and on PASTURE farm heifers spent either the day or day and night on pasture. [file peerj-08-9395-s001.pdf]

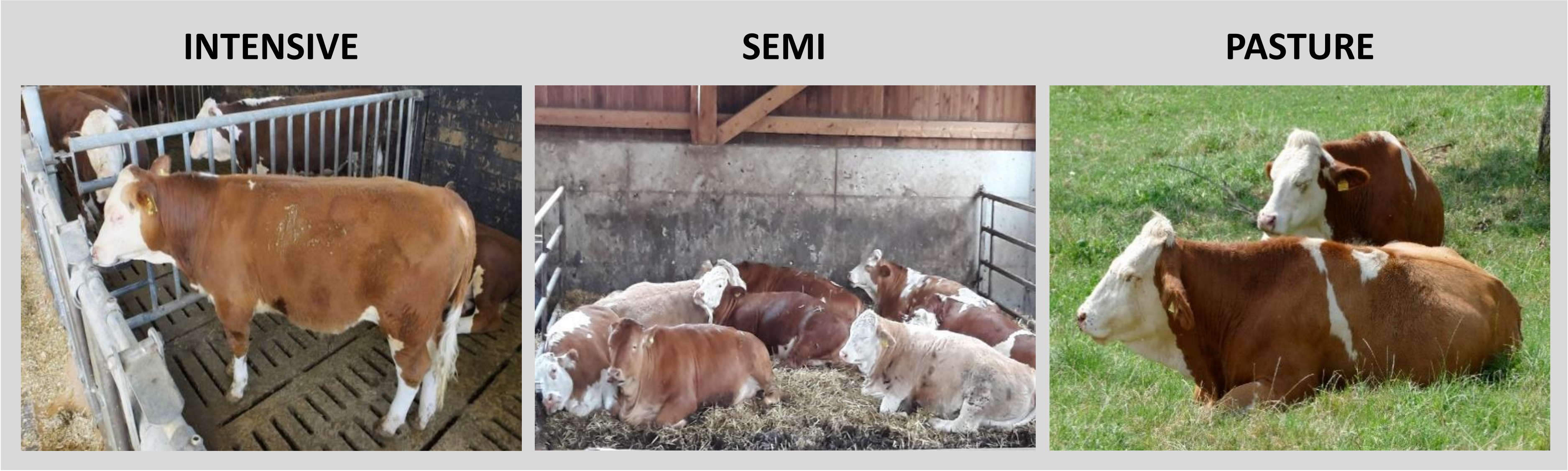

Figure 1. Pictures illustrating the three husbandry systems. INTENSIVE: heifers in pens with fully-slatted floor. SEMI: heifers in a straw-bedded lying area. PASTURE: heifers on pasture.
