# Supplementary material for: Doing nothing and what it looks like: inactivity in fattening cattle
Source: PeerJ. 2020 Jul 21;8:e9395. doi: 10.7717/peerj.9395 (PMC7512136; doi:10.7717/peerj.9395)
Supplement: Supplemental Information 2 — Note that some movements could be classified as both the animal still being inactive and the animal becoming active based on the number of times this movement was shown (up to two consecutive times: classified as still being inactive, more than two consecutive times: classified as becoming active). [file peerj-08-9395-s002.docx]

**SI Table 1.** Movements classifying standing and lying animals as being inactive or being active

|  | **Movements that classify the animal as being/remaining inactive** | **Movements that interrupt inactivity; the animal is recorded as being active** |
| --- | --- | --- |
| Body movements | - Maximum two steps forward or backward - Singe movement of one leg, including a kick after flies - Lying down or standing up without further movements - Stretching while standing or lying - Skin twitching - Urinating or defecating - Any tail movements | - Scratching self with one foot more than twice - Scratching self on barn equipment or objects on pasture - Licking self more than twice - Sniffing an object or conspecific |
| Head movements | - Head shaking (while standing or lying) - Snapping after flies by quickly throwing the head towards one side of the body - Ear movements - Eye blinks - Yawning - Coughing - Sneezing | - Flehming |
| Vocalisations | - Humming | - Mooing |
| Interactions with a conspecific | - Being licked without obvious reaction - Being nibbled without obvious reaction - Being mounted - Receiving a head butt without obvious reaction - Being displaced and being inactive thereafter | - Licking a conspecific - Nibbling on a conspecific - Mounting a conspecific - Head butting - Displacing a conspecific |

Note that some movements could be classified as both the animal still being inactive and the animal becoming active based on the number of times this movement was shown (up to two consecutive times: classified as still being inactive, more than two consecutive times: classified as becoming active).
