# Supplementary material for: Doing nothing and what it looks like: inactivity in fattening cattle
Source: PeerJ. 2020 Jul 21;8:e9395. doi: 10.7717/peerj.9395 (PMC7512136; doi:10.7717/peerj.9395)
Supplement: Supplemental Information 3 [file peerj-08-9395-s003.docx]

**SI Table 2.** Overview of some farm characteristics per husbandry system

|  | **INTENSIVE** | **SEMI** | **PASTURE** |
| --- | --- | --- | --- |
| Separate areas | no | yes: feeding area, activity area, lying area | structured by trees and location of the drinker(s) |
| Floor type | fully-slatted floor | deep-littered lying area and slatted or solid floor in feeding area | grass/soil |
| Straw bedding | no | yes | no |
| Outdoor run | no | no | n.a. |
| Number of animals per group | 5 – 10 | 4 – 14 | 9 – 31 |
| Space allowance per animal | 2.9 m^2^  (2.2 – 4.2 m^2^) | 3.9 m^2^  (2.9 – 5.5 m^2^) | ~ 1250 m^2^  (250 – 3800 m^2^) |
| Feeding system | total mixed ration | total mixed ration | grass plus total mixed ration (2 farms with day grazing only); grass only on the farm with continuous pasturing |
