# Supplementary material for: Doing nothing and what it looks like: inactivity in fattening cattle
Source: PeerJ. 2020 Jul 21;8:e9395. doi: 10.7717/peerj.9395 (PMC7512136; doi:10.7717/peerj.9395)
Supplement: Supplemental Information 4 — The mean percentage of time (some parts of) the focal animals were recorded as “Out of sight” with respect to the different categories of the Inactivity Ethogram is presented per husbandry system. [file peerj-08-9395-s004.docx]

|  | **INTENSIVE** | **SEMI** | **PASTURE** |
| --- | --- | --- | --- |
| Inactive/active | 0.4 | 0.5 | 4.1 |
| Lying postures | 1.8 | 2.6 | 0.3 |
| Head postures | 2.2 | 5.5 | 2.2 |
| Ear postures | 4.2 | 6.0 | 4.2 |
| Eye closure | 8.9 | 6.4 | 7.3 |
| Tail movement | 0.1 | 0.0 | 1.5 |

**SI Table 3.** “Out of sight” for the different categories of the Inactivity Ethogram
